# Plant-derived immunomodulators: an insight on their preclinical evaluation and clinical trials

**DOI:** 10.3389/fpls.2015.00655

**Published:** 2015-08-25

**Authors:** Ibrahim Jantan, Waqas Ahmad, Syed Nasir Abbas Bukhari

**Affiliations:** Drug and Herbal Research Centre, Faculty of Pharmacy, Universiti Kebangsaan Malaysia Kuala Lumpur, Malaysia

**Keywords:** immunomodulation, curcumin, resveratrol, epigallocatechol-3-gallate, quercetin, colchicine, capsaicin

## Abstract

The phagocyte–microbe interactions in the immune system is a defense mechanism but when excessively or inappropriately deployed can harm host tissues and participate in the development of different non-immune and immune chronic inflammatory diseases such as autoimmune problems, allergies, some rheumatoid disorders, cancers and others. Immunodrugs include organic synthetics, biological agents such as cytokines and antibodies acting on single targets or pathways have been used to treat immune-related diseases but with limited success. Most of immunostimulants and immunosuppressants in clinical use are the cytotoxic drugs which possess serious side effects. There is a growing interest to use herbal medicines as multi-component agents to modulate the complex immune system in the prevention of infections rather than treating the immune-related diseases. Many therapeutic effects of plant extracts have been suggested to be due to their wide array of immunomodulatory effects and influence on the immune system of the human body. Phytochemicals such as flavonoids, polysaccharides, lactones, alkaloids, diterpenoids and glycosides, present in several plants, have been reported to be responsible for the plants immunomodulating properties. Thus the search for natural products of plant origin as new leads for development of potent and safe immunosuppressant and immunostimulant agents is gaining much major research interest. The present review will give an overview of widely investigated plant-derived compounds (curcumin, resveratrol, epigallocatechol-3-gallate, quercetin, colchicine, capsaicin, andrographolide, and genistein) which have exhibited potent effects on cellular and humoral immune functions in pre-clinical investigations and will highlight their clinical potential.

## Introduction

Immunity is the body’s natural defense system against various infectious diseases. The factors which trigger immunity include previous infection, immunization, and various external stimuli (Baxter, 2007). Besides, immunity is capable of discriminating among body’s own proteins/cells and foreign entities. As soon as the foreign particle is identified, the collective and coordinated response of specific cells and mediators against strange substances constitutes the immune response (Baxter, 2007). Based on the function, immune system has been categorized in two broad categories, i.e., innate immune system (non-specific immune system) and adaptive immune system (specific or acquired immune system; Vesely et al., 2011). The microbiological, chemical and physical barriers are also sometimes included in innate immunity, however, the main mediators of immune system which deliver instant defense include cytokines, acute phase proteins, macrophages, monocytes, complement, and neutrophils. Various distinct moieties expressed by pathogens, known as pathogen-associated molecular patterns (PAMPs), are recognized by host to detect presence of a pathogen. The germline-encoded and evolutionarily conserved host sensors known as pattern recognition receptors (PRRs) recognize the PAMPs. Once the PRRs recognize the PAMPs, an array of immune responses are quickly triggered via induction of different type I interferons, chemokines, and cytokines. An important role in host’s defense is played by PRRs families such as DNA receptors (cytosolic sensors for DNA), NOD-like receptors, RIG-I-like receptors and toll-like receptors (Parkin and Cohen, 2001). All phases of non-specific immunity include antigen-presenting cells and macrophages which play pivotal roles in antibody-dependent cell-mediated cytotoxicity, secretion of cytokines, nitric oxide (NO) production and antigen presentation, processing and phagocytosis. Dendritic cells are responsible for the activation of naïve and memory B and naïve T cells. During various phases of dendritic cells’ differentiation, the effectors of innate immunity including natural killer (NK) cells are regulated, which govern specific and natural immune responses by producing tumor necrosis factor-α (TNF-α), interferon-γ (IFN-γ) and granulocyte–macrophage colony-stimulating factor (GM-CSF; Moradali et al., 2007). Complement system is the tertiary relevant component of innate immunity. The complement system is the humoral immunity’s main effector among all the physiological systems of host defense (Oh et al., 2000). C3a and C3b (complement system’s components) are activated by C9, and amplify and mediate immune response.

**FIGURE 1:**
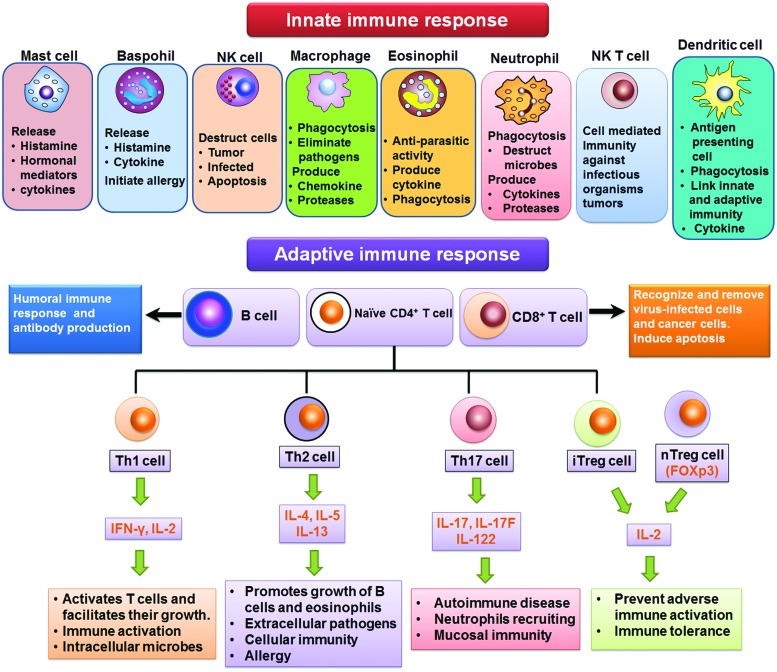
**Cells of innate and adaptive immune response**.

Adaptive immunity is acquired by generating pathogen (antigen)-specific B and T lymphocytes through a gene rearrangement process. The exposure of body to antigen with aim to produce adaptive immune reaction that develops in weeks/months but may last through whole life is called active immunity. The active immunity may either be acquired or natural. The immune system of higher animals is equipped with adaptive immunity. The antigen specific reactions (via B and T lymphoctyes) are involved in adaptive immunity. The strong phagocytic action of myeloid cells and cytotoxic T lymphocytes is enhanced by Th1 lymphocytes which produce TNF-α, IFN-γ, and IL-2. The IL-4, IL-5, and IL-10 are produced by Th2 lymphocytes (which are the mediators of humoral immunity), categorized by B lymphocytes-mediated production of antibodies. The toxins or microorganisms are neutralized after binding with the antibodies. Moreover, antibodies have the ability to opsonize different pathogens, immobilize bacteria, and trigger microorganisms’ destruction by phagocytes via activation of complement proteins (Spellberg and Edwards, 2001). The cell types involved in innate and adaptive immune responses are summarized in **Figure 1**.

## Immunomodulators

In healthy organism, the immune system maintains homeostasis within the body. The function and efficiency of the immune system are influenced by various exogenous and endogenous factors resulting in either immunosuppression or immunostimulation. Several agents possessing an activity to normalize or modulate pathophysiological processes are called immunomodulators (Puri et al., 1994). The biomolecules of synthetic or biological origin capable of modulating, suppressing and stimulating any components of adaptive or innate immunity are known as immunomodulators, immunorestoratives, immunoaugmentors, or biological response modifiers. Immunomodulators are generally categorized into immunoadjuvants, immunostimulants, and immunosuppressants in clinical practice. Immunoadjuvants are specific immune stimulators which enhance the efficacy of vaccine. Agents that activate or induce the mediators or components of immune system are called as immunostimulants. The resistance against autoimmunity, cancer, allergy, and infection is enhanced by immunostimulants. On the other hand, immunosuppressants are the molecules that inhibit the immune system, can be used to control the pathological immune reaction subsequent to organ transplantation. Additionally, these agents can also be used in the treatment of infection-associated immunopathology, hypersensitivity reactions, and autoimmune diseases. A number of monoclonal antibodies and chemically synthesized compounds are also being used as immunomodulators. However, there are major limitations to the general use of these agents which are summarized in **Table 1**. Therefore, immunomodulatory entities with additional safety and effectiveness are still in need. Due to the occurrence of chemical drugs-related adverse effects, natural immunomodulators are the potential agents to replace them in therapeutic regimens.

**Table 1:** Mechanism of action and side effects of immunomodulatory agents.

Immunomodulators	Category	Example	Mechanism	Main side effects	Reference
Immunosuppressant	Inhibitors of lymphocyte gene expression.	Glucocorticoids	Decreased leukocytes extravasation. Reduce pro-inflammatory cytokines expression.	Growth retardation in children, bone necrosis, osteopenia, hyperglycemia, and hypertension.	Golan (2008), Jain (2008)
	Inhibitors of lymphocyte signaling	Cyclosporine	Antigen-triggered signal transduction constrain in T lymphocytes. Reduce expression of lymphokines and anti-apoptotic proteins.	Renal dysfunction, gum hyperplasia, hyperuricemia, hyper-cholesterolemia, diabetogenic.	Jain (2008), Tortora and Derrickson (2008)
		Tacrolimus	Inhibits T-cell activation by inhibiting calcineurin.	Nephrotoxicity, neurotoxicity hypertension, hyperkalemia, hyperglycemia.	Singhal (2007), Sengupta (2009)
		Sirolimus	Inhibit activation and proliferation of T-lymphocyte. Down regulate T-cell growth factor receptor and IL-2.	Level of serum cholesterol and triglycerides increase. Impaired renal functions, prolong delayed graft function.	Katzung and Trevor (2009), Gilman’s (2008)
	Cytotoxic agents	Azathioprine, azathioprine sodium	Inhibit *de novo* synthesis of purine which leads to inhibit proliferation of lymphocytes.	Leukopenia, thrombocytopenia, hepatotoxicity, alopecia, GI toxicity, pancreatitis.	Katzung and Trevor (2009)
		Mycophenolatmofetil	Inhibit *de novo* synthesis of guanine by inhibiting inosine monophosphate dehydrogenase.	Leukopenia, diarrhea, vomiting, sepsis associated with cytomegalovirus.	Rang and Dale (2007), Richard and Pamela (2009)
	Alkylating agent	Cyclophosphamide	Prevent the cell division and protein synthesis by cross linking in the strands of DNA.	Pancytopenia and hemorrhagic cystitis, graft versus–host disease syndrome, cardiac toxicity, electrolyte disturbances	Mythili et al. (2004)
	Cytokine inhibitors	Etanercept, infliximab, adalimumab, anakinra, daclizumab, basiliximab	Bind with tumor necrosis factor-alpha and inhibit TNFα to bind with TNFα receptors.	Reactivation of tuberculosis, psoriasis, invasive fungal infections, hypersensitivity, and anaphylaxis.	Keane et al. (2001), Baudouin et al. (2003), Bartelds et al. (2011)
	Antibodies against specific immune cell molecules	Antithymocyte globulin	Reduce circulating lymphocytes by inducing cytotoxicity. To inhibit lymphocytes functions, it binds with cell surface molecules which regulate cell functions.	Fever, chills, hypotension, serum sickness, glomerulonephritis, leukopenia, and thrombocytopenia.	Hansel et al. (2010)
		Muromunab	Prevent subsequent antigen recognition by causing internalization of the T-cell receptor.	Cytokine release syndrome, high fever, chills/rigor, myalgias, arthralgias, aseptic meningitis, cardiovascular collapse, cardiac arrest.	Sgro (1995), Katzung and Trevor (2009), Liu and Li (2014)
	Intercellular adhesion molecules inhibitors	Efalizumab	Inhibit T-cell adhesion and trafficking by inhibiting LFA-1-ICAM interaction.	Bacterial sepsis, viral meningitis, invasive fungal disease, and progressive multifocal leukoencephalopathy.	Berger et al. (2009)
Immunostimu-lants	Imidazothiazole derivative	Levamisole	Repair the suppressed immune function of B and T lymphocytes, monocytes, and macrophages.	Flu-like symptoms, allergic manifestation, nausea, and muscle pain.	Auffenberg et al. (2013)
	Recombinant cytokines	Aldesleukin, interferon alpha, interferon gamma.	Inhibit cell proliferation and enhance immune cells activities such as increased macrophages phagocytosis and T lymphocytes cytotoxicity.	Hypotension, arrhythmias, cardiomyopathy myocardial infarction, GI distress, and anorexia.	Katzung and Trevor (2009), Strayer and Carter (2012)
	Hormonal analog	Isoprinosine	Enhance cytokines (i.e., IL-1, IL-2, and IFN-γ) production. It induces lymphocytes proliferation.	CNS depressant, transient nausea, and increase uric acid level in serum and urine.	Campoli-Richards et al. (1986), Parnham and Nijkamp (2005)

Presently, majority of research and development still focuses on biochemicals, biologics, or single compounds as lead compounds that aim at particular targets linked with a disease. It is difficult to attain single compound chemicals with high selectivity and potency, and low toxicity for targeted molecular/cellular targets and diseases. Hence, the design and development of drug candidates from numerous conventional or complementary and alternative medicines is gaining interest. The prevention and treatment of disease using plant-based medicines has been reported in human history. In all cultures and through all ages different parts of a huge number of plants were used as drugs against all kinds of ailments. Vinblastine, vincristine and their semi-synthetic derivatives isolated from the Madagascar periwinkle (*Catharanthus roseus*), capsaicin from chili peppers (*Capsicum* species), paclitaxel from Pacific yew (*Taxus brevifolia*) and galantamine from the Caucasian snowdrop (*Galanthus caucasicus*) are examples of medicines based on plant compounds. The plant-based compounds that served as lead structures and/or were chemically altered are dicoumarol (warfarin), artemisinin (artemether), camptothecin (topotecan and irinotecan), morphine (scores of derivatives), and salicylic acid (acetylsalicylic acid; Oberlies and Kroll, 2004).

Recently the clinical potential of six plant-derived antiinflammatory compounds: curcumin, colchicine, resveratrol, capsaicin, epigallocatechin-3-gallate (EGCG), and quercetin has been highlighted in Fürst and Zündorf (2014). The present review will give an overview of these widely investigated plant-derived compounds including andrographolide and genistein, which have exhibited potent effects on cellular and humoral immune functions in pre-clinical investigations and will highlight their clinical potential.

**FIGURE 2:**
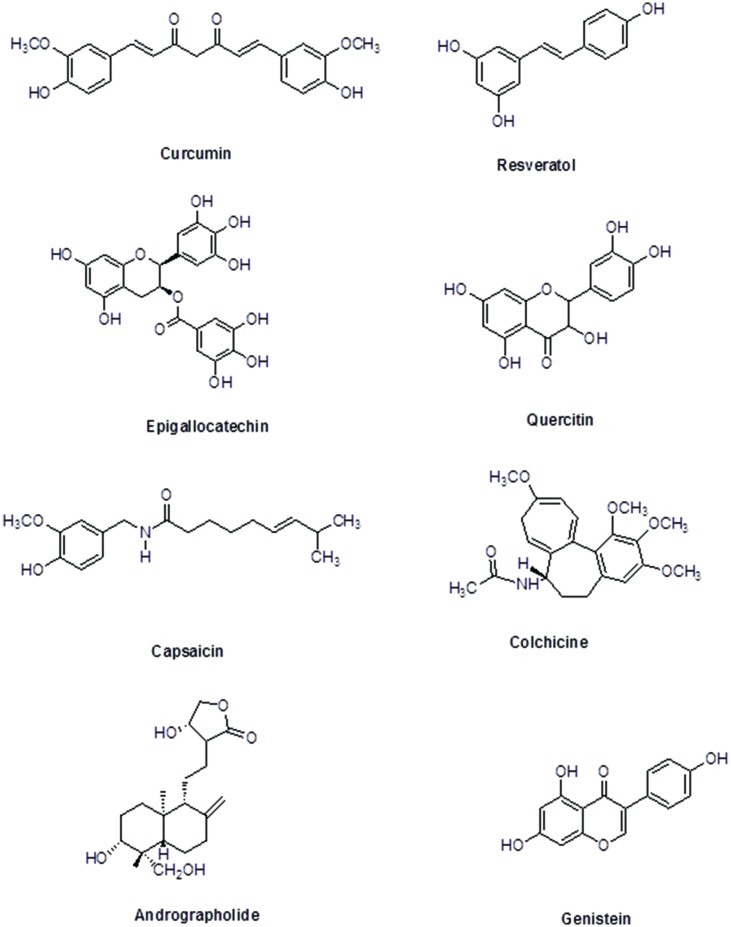
**Chemical structures of selected plant-derived immunomodulators in clinical trials**.

The immunomodulatory characteristics of plant-based therapeutics have gathered attention of researchers. Innovative technologies and the excessive research on immunomodulatory natural products, plants, their extracts, and their active moieties with immunomodulatory potential, may provide us with valuable entities to develop as novel immunomodulatory agents to supplement the present chemotherapies. This review focuses on the plant-based immunomodulatory compounds undergoing clinical trials. Additionally, the potential use as immunomodulatory agents, modes of action and plant origin of numerous important plant-based lead compounds has also been comprehensively discussed (**Figure 2**). Beside these compounds, other phytochemicals including essential oils, steroids, terpenoids, phenolics, pigments, flavonoids, and alkaloids, etc. have exhibited worth mentioning immunomodulatory effect. Plant-derived compounds showing promising potential as immunomodulatory agents are listed in **Table 2**.

**Table 2:** Plant derived immunomodulatory agents.

Chemical class	Plant source	Mechanism	Reference
**Alkaloids**			
Berberine	*Coptis chinensis* Franch	Down-regulate T-helper cells cytokines [Th1 (TNF-α, IL-2), and Th2 (IL-4)] production.	Lin and Lin (2011)
Chelerythrine	*Chelidonium majus* L.	Inhibit PGE_2_ release by regulating cyclooxygenase-2 activity	Niu et al. (2011)
Gelselegine	*Gelsemium elegans*	Inhibited T lymphocyte proliferation.	Xu et al. (2012)
Pseudocoptisine	*Corydalis turtschaninovii* Besser	Suppressing the phosphorylation of ERK and p38 to inhibit NF-κB activation, which resulted in reduction of pro-inflammatory mediators level (iNOS, COX-2, TNF-α, and IL-6).	Yun et al. (2009)
Leonurine	*Leonurus japonicas* Houtt	Downregulate TNF-α, IL-6, iNOS, and COX-2 and upregulate IL-10 by inhibiting the expression of toll like receptors and the activation of NF-κB. Inhibit the ICAM-1, VCAM-1 activity.	Liu et al. (2012), Song et al. (2015)
Piperine	*Piper longum* Linn	Reduce level of pro inflammatory cytokines IL-1β, IL-6, and TNF-α. Down regulate expression of COX-2, NOS-2, and NF-κB. Inhibit eicosanoide generation by inhibiting phospholipase A_2_ and TXA_2_ synthase activity.	Vaibhav et al. (2012), Son et al. (2014)
Sinomenine	*Sinomenium acutum* (Thunb.) Rehd.etWils	Inhibit the production and release of inflammatory cytokine. Inhibit expression of VCAM-1.	Chen et al. (2011), Oh et al. (2012)
Koumine	*Gelsemium elegans*	Inhibit T lymphocyte proliferation.	Xu et al. (2012)
Lycorine	*Lycoris radiate*	Inhibit iNOS and COX-2 activity.	Kang et al. (2012)
Sophocarpine	*Sophora alopecuroides* L.	Inhibit production of NO and pro inflammatory cytokines TNF-α and IL-6. Inhibit expression of iNOS and COX-2.	Zhang et al. (2008), Gao et al. (2012)
Rhynchophylline	*Uncaria rhynchophylla* (Miq.) Jack	Inhibit phosphorylation of mitogen-activated protein kinases. Inhibit production of pro inflammatory cytokines, NO, PGE_2_, monocyte chemoattractant protein MCP-1, TNF-α, IL-1β.	Song et al. (2012)
Tetrandrine	*Stephania tetrandra*		Wu et al. (2011)
Matrine	*Sophora flavescens* Ait	Reduced production of reactive oxygen species inflammatory mediators. Inhibit myeloperoxidase and maleic dialdehyde activity.	Zhang et al. (2011)
**Essential oils**			
Z-ligustilide	*Angelica sinensis* (Oliv.) Diels	Inhibit iNOS and COX-2 induction by regulating the NF-kB and MAPK signal pathways.	Chung et al. (2012)
Tetramethylpyra-zine	*Ligusticum chuanxiong* Hort	Inhibit pro inflammatory cytokines and reactive oxygen species production. Inhibit macrophages chemotaxis, neutrophile infiltration, and nitric oxide synthase activity. Block the phosphorylation of p38 mitogen-activated protein kinase.	Liu et al. (2010), Hu et al. (2013)
**Flavonoids**			
**Chalcone**			
Butein	*Semecarpus anacardium, Dalbergia odorifera, Toxicodendron vernicifluum*	Suppress NO production by attenuating iNOS expression. Inhibit translocation of NF-κB.	Wang et al. (2014)
Xanthohumol	*Humulus lupulus*	Inhibit NO production which is induced by LPS and INF-γ.	Zhao et al. (2003)
Dihydroxanthohumol	*Humulus lupulus*	Inhibit NO production which is induced by LPS and INF-γ.	Zhao et al. (2003)
Mallotophilippens C, D, E	*Mallotus, Philippinensis*	Inhibit mRNA gene expression of iNOS, COX-2, IL-6, and IL-1β. Inactivate NF-κB.	Daikonya et al. (2004)
Licochalcone E	*Glycyrrhiza inflata*	Inhibit secretion of pro-inflammatory cytokines IL-1β, IL-6, and TNF-α by inhibiting the activity of NF-κB and activator protein (AP-1).	Lee et al. (2013a)
**Flavones**			
Luteolin	*Lonicera* *Japonica*	Decreased secretion of inflammatory mediators (INF-γ, IL-6) reduced COX-2, ICAM-1 expression. Block heat shock protein 90 activity.	Chen et al. (2007, 2014), Ziyan et al. (2007)
Apigenin	*Cynodon dactylon, Salvia officinalis* L*., Portulaca oleracea, Mentha longifolia*	Downregulate expression of cytokines (IL-1α, TNF-α, IL-8). Decreased response of Th1 and Th17 cells. Downregulate the expression of COX-2 and iNOS. Decreased expression of ICAM and VCAM leading to decreased neutrophile Chemotaxis.	Nicholas et al. (2007), Kang et al. (2009)
Chrysin	*Picea crassifolia*	Inhibited production of pro-inflammatory cytokine (TNFα, IL-1β, and IL-6). By modulation of intracellular calcium reduce histamine release from mast cells.	Hougee et al. (2005), Shin et al. (2009), Bae et al. (2011)
Nobiletin	*Citrus nobilis* Lour, *Citrus aurantium* L.	Inhibit pro-inflammatory mediators, COX-2 and iNOS expression by blocking NF-κB and MAPK signaling pathway.	Kang et al. (2011)
Baicalein	*Scutellaria altissima* L.	Inhibit mRNA expression of iNOS, COX-2, and TNF-α. Inhibit production of NO and inflammatory cytokine (IL-1β, PGE_2_, and TNF-α) by regulating NF-κB and ER-dependent pathway. Inhibit adhesion and migration of leukocytes by inhibiting cell adhesion molecules expression.	Chandrashekar et al. (2012), Fan et al. (2013), Lee et al. (2015)
Oroxylin A	*Scutellariae baicalensis* Georgi	Inhibit NO production and iNOS and COX-2 proteins expression of via inhibiting nuclear factor-κB pathway. Enhance antioxidant response element-luciferase reporter activity by increasing the expression of nuclear factor erythroid 2-related factor 2 proteins.	Chen et al. (2000), Li et al. (2007), Ye et al. (2014)
Wogonin	*Scutellaria baicalensis* Georgi	Inhibit adhesion and migration of leukocytes by inhibiting cell adhesion molecules expression. Reduces allergic airway inflammation by inducing eosinophil apoptosis through activation of caspase-3.	Guo et al. (2007), Lee et al. (2015), Lucas et al. (2015)
**Flavonols**			
Quercetin	*Dysosma veitchii* Hemsl. et Wils	Decreased expression of pro-inflammatory cytokines, NF-κB, and iNOS. Reduce expression of VCAM-1 and E-selectin.	Shaik et al. (2006), Kleemann et al. (2011), Choi et al. (2012)
Kaempferol	Found in various fruits and vegetables e.g., tea, tomato, cruciferous vegetables, apple, etc.	Reduce iNOS and COX-2 activity through suppression of the signaling of STAT-1, NF-kappa B, and AP-1. Decrease the gene expression of ICAM-1, VCAM-1, and monocyte chemotactic protein-1 (MCP-1).	Hamalainen et al. (2007), Kong et al. (2013)
Rutin	*Ruta graveolens*	Inhibit leukocyte migration. Suppress production of TNF-α and IL-6. Inhibit activation of NF-κB and extracellular regulated kinases.	Yoo et al. (2014)
**Flavanols**			
Epigallocatechin-3-gallate	*Camellia sinensis* L.	Inhibit reactive oxygen species generation, MAPKs phosphorylation, adhesion molecules expression signal transducers and activators of transcription 3 (STAT-3) and activating transcription factor 2 translocation through induction of heme oxygenase-1 and suppressors of cytokine signaling -3 expression.	Lee et al. (2013b)
**Isoflavones**			
Daidzein	*Pueraria mirifica, Pueraria lobata, Glycine max*	Decreases TNF-α, IL-1β, MCP-1, NO, and iNOS expression at mRNA level.	Hamalainen et al. (2007)
Genistein	*Glycine max*	Inhibited expression of iNOS and COX-2. Decreased IL-1β and TNF-α production via activation of PPARs.	Valles et al. (2010)
Puerarin	*Pueraria lobata* (wild) Ohwi	Decrease cytokines level. Inhibit NF-κB and activation of signal transducers and activators of transcription 3 (STAT3).	Liu et al. (2013)
**Phloroglucinols**			
Myrtucommulone	*Myrtus communis* L.	Inhibit the PGE_2_ production by inhibiting the mPGES-1 activity without significantly inhibiting the COX enzymes activity.	Koeberle et al. (2009)
Arzanol	*Helichrysum italicum*	Reduce eicosanoids generation by inhibiting lipooxygenase and cyclooxygenase activity in arachidonic acid metabolism pathway.	Rossi et al. (2009), Bauer et al. (2011)
**Quinones**			
Thymoquinone	*Nigella sativa* L.	Inhibited LPS-induced fibroblast proliferation and H_2_O_2_-induced 4-hydroxynonenal generation. Inhibit IL-1β, TNF-α, MMP-13, COX-2, and PGE_2_ while blocking phosphorylation of MAPK p38, ERK1/2, and NF-kBp65.	Vaillancourt et al. (2011)
Shikonin	*Lithospermum erythrorhyzon* Sieb. etZucc.	Inhibit NF-κB activity, inhibit Th1 cytokines expression and induce Th2 cytokines.	Andújar et al. (2010)
Emodin-8-*O*-β-D glucoside	*Polygonum amplexicaule* D. Don var. *sinense Forb.*	Stimulate proliferation and differentiation of osteoblasts. Inhibit PGE2 production by increased alkaline phosphatase expression in MC3T3-E1.	Xiang et al. (2011)
**Other**			
Apocynin	*Apocynum cannabinum* L. (Canadian hemp), *Picrorhiza kurroa* Royle ex Benth.	Inhibit NADPH oxidase activity. Suppress pro-inflammatory cytokines and CD4+ and CD8+T cells production.	Stefanska and Pawliczak (2008), Kim et al. (2012)
**Stilbenes**			
Resveratrol	*Fallopia japonica*, grape, nuts	Decrease MPO activity and mPGES-1 to basal levels. Inhibit iNOS and COX-2 expression. Reduce the pro-inflammatory cytokines IL-8, TNFα, IFN-γ, and IL-1α. Increase the anti-inflammatory cytokine IL-10 level.	Youn et al. (2009), Vang et al. (2011)
Piceatannol	*Fallopia japonica*, grape, nuts	Decrease iNOS expression. Inhibit transcription factors activation such as NF-kB, ERK, and STAT3.	Youn et al. (2009), Vang et al. (2011)
**Terpenoid**			
14-deoxyandrographolide	*Andrographis paniculata*	Enhanced proliferation of lymphocytes. Enhanced IL-2 induction in lymphocytes.	Kumar et al. (2004)
14-deoxy-11,12-didehydroandrographolide	*Andrographis paniculata*	Enhanced proliferation of lymphocytes. Enhanced IL-2 induction in lymphocytes.	Kumar et al. (2004)
Ginsan	*Panax ginseng*	Enhances the production of cytokines and reactive oxygen species by macrophages. Stimulates the phagocytic activity of macrophages.	Shen et al. (2002), Song et al. (2002)
Oleanolic acid	*Luffa cylindrica*, *Phytolacca americana*	Reduce level of IL-1α, IL-6, and TNF-α, as well as their effect on complement pathway though the inhibition of C3 convertase. Inhibits adenosine deaminase activity.	Brinker et al. (2007)
Echinocystic acid	*Luffa cylindrica*	Enhance phagocytic index of macrophages in humoral and cell-mediated immune responses.	Khajuria et al. (2007)
Triptolide	*Tripterygium wilfordii*	Inhibits lymphocyte activation and pro-inflammatory cytokines gene expression (IL-2, iNOS, TNF-α, COX-2, and IFN-γ). It also inhibits activation of transcription factors such as NF-kB, NFAT, and STAT3.	Brinker et al. (2007)
Demethylzelasteral	*Tripterygium wilfordii*	Inhibits proliferation of vascular endothelial cells.	Ramgolam et al. (2000)
Celastrol	*Tripterygium wilfordii*	Inhibit expression of pro-inflammatory cytokines, adhesion molecules, proteasome activity, and topoisomerase II.	Kannaiyan et al. (2011)
Asiaticoside	*Centella asiatica*	Decrease NO production.	Hartog et al. (2009)
Madecassoside	*Centella asiatica*	Reduce spleen cells proliferation. Inhibition of pro-inflammatory mediators such as TNF-α and IL-6. Inhibit production of PGE_2_, and COX-2.	Li et al. (2009)
11-keto-β-boswellic acid	*Boswellia carteri*	Decrease pro-inflammatory cytokines such as IL-1, IL-2, IL-4, IL-6, and IFN-γ through inhibition of NF-kB activation.	Ammon (2006)

## Selected Immunomodulatory Plant-Derived Single Chemical Entities in Clinical Trials

### Curcumin

Curcumin is a natural diarylheptanoid compound found in the rhizome of *Curcuma longa* and related species. The medicinal benefits of curcumin have been known since centuries. A variety of biological and pharmacological properties of curcumin has been reported including anti-cancer, anti-oxidant, anti-angiogenic, anti-proliferative, pro-apoptotic, etc. Curcumin is one of the most extensively studied compound for its immunomodulatory properties. Curcumin lessened inflammatory responses by inhibiting NO production, cyclooxygenase-2 (COX-2), nuclear factor-kappa B (NK-kB), inducible nitric oxide synthase (iNOS), and lipoxygenase in NK cells and IFN-γ, or TNF-α activated macrophages (Bhaumik et al., 2000; Surh et al., 2001). In phorbol 12-myristate 13- acetate (PMA) and H_2_O_2_-stimulated human myelomonoblastic cell line, curcumin inhibited NF-κB activation via prevention of degradation and phosphorylation of I kappa B alpha (IκB-α; Singh and Aggarwal, 1995). Protein kinase C, which regulates the proliferation and survival of cell, is activated by PMA. Additionally, LPS and TNF-α also activate protein kinase C, which then activates NF-κB (Holden et al., 2008). Therefore, curcumin might weaken NF-kB activation by the inhibition of protein kinase C. The anti-inflammatory activity of curcumin was partially mediated by inhibiting activator protein (AP)-1 and transcription factor NF-κB. NF-κB and AP-1 act collectively and may enhance tumor development. The treatment of glioma cells with 25 μM curcumin reduced the binding of NF-κB and AP-1 (Dhandapani et al., 2007). The AP-1 activation was also suppressed by curcumin in TNF-activated bovine aortic endothelial cells (Xu et al., 1997). The activated immune cells release pro-inflammatory cytokines that play key role in inflammation. The expression of pro-inflammatory cytokines TNF-α, IL-1, IL-6, and IL-12 was inhibited by curcumin via LPS- or PMA-stimulated monocytes, macrophages, dendritic cells, and splenic lymphocytes (Gao et al., 2004; Kim et al., 2005). The attachment of T cells to endothelial and antigen presenting cells is dependent on cell adhesion molecules. The attachment of monocytes to endothelial cells was blocked by pre-treatment with curcumin. Moreover, the expression of intracellular adhesion molecule (ICAM)-1, vascular cell adhesion molecule (VCAM)-1, and endothelial leukocyte adhesion molecule (ELAM)-1 was also decreased in TNF-α-stimulated human umbilical vein endothelial cells (HUVECs) via inhibition of NF-κB (Kumar et al., 1998).

Jurenka (2009) reviewed pre-clinical and clinical trials of curcumin that were carried out at different places. Presently, 116 studies regarding the diverse actions of curcumin could be found on http://www.clinicaltrials.gov/. Among these 99 studies were based on the anti-inflammatory properties of curcumin. The most noticeable diseases for which trials had been conducted were cancer (e.g., lung, prostate, breast, pancreatic, and colorectal), rheumatoid arthritis and inflammatory bowel diseases (IBD; ulcerative colitis and Crohn’s disease), which reflect the pleiotropic actions of curcumin. This information shows that curcumin is still under extensive clinical investigation. Future clinical trials will predominantly deal with still ongoing various types of cancer and the action of curcumin on cognitive damage. Additionally, the inflammatory conditions will still be studied. Curcumin often acts as an adjunct treatment or dietary supplement to the standard therapy in these trials. The review on effects of curcumin in ulcerative colitis (Kumar et al., 2012) stated that curcumin, when given as adjunct therapy may be effective and safe therapy for the maintenance of remission in quiescent ulcerative colitis. Nonetheless, more operationally thorough randomized controlled trials are required. This conclusion from the authors reflects the overall situation. However, Fürst and Zündorf (2014) suggested that these are preliminary clinical trials which are frequently too weak and of low quality to draw a conclusion due to the low number of enrolled patients, which normally ranges from 10 to 30. As suggested by the authors, more operationally thorough and serious randomized controlled trials are required to evaluate the compound as an effective and safe agent for human use. It is worth mentioning that curcumin suffers from its low bioavailability, though substantial improvement has been made to address this issue via chemical and technological methods (Anand et al., 2007). The future of curcumin as an approved option for the prevention or treatment of the above mentioned indications relies on the findings of high-quality and big cohort studies in the future. Still, from the available literature, a conclusion can be drawn that curcumin exhibits good safety profile; it is nontoxic and well tolerated.

### Resveratrol

Resveratrol, chemically (5-[(E)-2-(4-hydroxyphenyl) ethenyl] benzene-1,3-diol), is a derivative of stilbene and phytoalexin. It is found in various dietary products and plants including grapevines, red wine and peanuts. Similar to curcumin, resveratrol was found to exert a variety of pharmacological activities such as antimicrobial, chemopreventive, anticancer/proapoptotic, anti-inflammatory, and antioxidant properties. The inflammatory molecules are strongly inhibited by resveratrol. The immunomodulatory activities of resveratrol include the inhibition of NF-κB in PMA, LPS or TNF-α-mediated macrophages, epithelial (HeLa), Jurkat, myeloid (U-937) and dendritic cells. NF-κB activation was inhibited by resveratrol via inhibition of IκB kinase (Holmes-McNary and Baldwin, 2000; Manna et al., 2000; Gao et al., 2001). The expression of COX-2 and iNOS in cytokine (IFN, IL-1, or TNF-α) stimulated human primary airway epithelial cells was also inhibited by resveratrol (Donnelly et al., 2004) whereas it also blocked the transcription of COX-2 in human mammary epithelial cells stimulated by PMA. The secretion of NO and TNF-α in LPS-activated N9 microglial and cortical microglia cells was also substantially suppressed by resveratrol (Bi et al., 2005), moreover the production of IL-12, IL-6, IL-1, TNF-α, and IFN-γ and by splenic macrophages and lymphocytes was also inhibited (Kowalski et al., 2005). Resveratrol also showed strong inhibition of *in vivo* C5 anaphylatoxin (C5a)-mediated inflammation. The release of inflammatory cytokines (MIP-1, IL-6, IL-1, and TNF-α) in C5a-activated mouse and human neutrophils was inhibited by the pre-incubation with resveratrol (10–40 M). In addition, ERK-phosphorylation, release of glucuronide and C5a induced oxidative burst (superoxide anion production) were also inhibited by resveratrol. Furthermore, resveratrol inhibited production of inflammatory cytokines and C5a-stimulated neutrophil recruitment/migration in C5a-stimulated acute peritonitis mouse model (Issuree et al., 2009). The expression of cell adhesion molecules was also found to be inhibited by resveratrol. The IL-6-stimulated ICAM-1 expression in endothelial cells was also decreased by resveratrol (Wung et al., 2005), in addition to the inhibition of *Porphyromonas gingivalis* LPS-induced endothelial dysfunction in human microvascular endothelial cells. Besides, ICAM-1 and VCAM-1 expression on human microvascular endothelial cells was blocked by inhibition of NF-κB activation (Park et al., 2009).

Based on the above-mentioned findings, a lot of emphasis was put into the explanation of the underlying mechanisms of action. Still, this enormous knowledge has yet not been translated into anapproved clinical medicine. More than 40 clinical trials on resveratrol have been listed in PubMed for a variety of diseases like coronary artery disease, obesity, diabetes, and so forth. The main focus of these studies was to either analyze inflammation-related parameters in blood cells (e.g., transcription factors or activated kinases) and in plasma (e.g., IL-6, IL-1β, TNF-α) or to report on functional parameters like the status of the endothelium (Bakker et al., 2010; Tome-Carneiro et al., 2013). Several trials persuasively showed that these parameters are usefully influenced by resveratrol. Though, whether this improved inflammatory status of the patients indeed leads to a clinically relevant improvement of the severity of the diseases or, most significantly, in a decreased incidence of disease-specific life-threatening events has not been studied. Currently 99 studies are listed on http://www.clinicaltrials.gov/, 49 studies have been completed while 28 clinical trials on resveratrol are currently recruiting or planned. Among them 27 studies are linked with the anti-inflammatory properties of resveratrol. The main field of interest is metabolic syndrome/type 2 diabetes, followed by mild cognitive impairment, non-alcoholic fatty liver disease, and polycystic ovary syndrome. Research would benefit by carrying out interventional studies with definite primary outcomes reflecting the stage or/and occurrence of the diseases on a long-term basis.

### Epigallocatechin-3-gallate

One of the most active and abundant polyphenol of green tea *Camellia sinensis* (Theaceae) is epigallocatechin-3-gallate (EGCG), chemically [(2R,3R)-5,7-dihydroxy-2-(3,4, 5-trihydroxyphenyl)-3,4-dihydro-2H-chromen-3-yl]3,4,5-trihydroxybenzoate]. It has been widely reported for its *in vitro* and *in vivo* chemopreventive, anti-angiogenic, anti-invasive, anti-proliferative, anti-inflammatory, and anti-oxidant effects (Singh et al., 2011; Yang et al., 2011b). Studies have showed that EGCG blocked the activation of NF-κB by inhibiting the degradation of IκBα (Muraoka et al., 2002) and also inhibited the MAPK pathways (Chung et al., 2001). The down-regulation of iNOS transcription and NO production in macrophages is dependent on the inhibition of NF-κB. In comparison, it was shown that EGCG improved the production of NO to inhibit endothelial exocytosis and leukocyte adherence to endothelial cells (Yamakuchi et al., 2008). Moreover, it was reported that EGCG blocked NF-κB activation in human endothelial cells and thus inhibited the expression of MCP-1 (Hong et al., 2007). By reducing mRNA expression of bax and caspase3 and by decreasing caspase3 activity, EGCG also decreased apoptosis (Hara et al., 2006; Park et al., 2006; Yu et al., 2007). Additionally, it inhibited expression of COX-2, proteasome dependent degradation, the MAPK pathway and growth factor-dependent signaling (e.g., of IGF-I, VEGF, and EGF; Yang et al., 2011a). Likewise, EGCG inhibited DNA methyltransferase 1, topoisomerase II and telomerase, thus affecting the functions of chromatin (Lee et al., 2005; Sadava et al., 2007; Bandele and Osheroff, 2008).

Surprisingly, though, notwithstanding encouraging pre-clinical results and the detailed mechanistic insights, clinical studies related to inflammation are lacking. A small scale study investigated the action of green tea and its extract on biomarkers of inflammation (e.g., ICAM-1, VCAM-1, IL-1β, IL-6, CRP, and adiponectin) in obese patients with metabolic syndrome. After 8 weeks treatment, green tea did not change the biomarker levels (Basu et al., 2011). Another clinical trial reported a useful impact of topical EGCG treatment on acne vulgaris, which may be in part due to anti-inflammatory effects (Yoon et al., 2013). A green tea extract was approved as a prescription drug in 2006 for the topical treatment of anal and genital warts (*Condylomata acuminate*). This key development fueled additional research to expand the indications of EGCG. Sixty-eight clinical trials are listed on http://www.clinicaltrials.gov/ for upcoming years. Fifteen studies are in recruiting stage while 33 studies have been completed. EGCG will be tested for its effects on albuminuria in diabetic nephropathy along with its action in patients with multiple-system atrophy, Huntington’s disease, fragile X syndrome, Down syndrome, Alzheimer’s disease (early stage), muscular dystrophy of the Duchenne type, and cardiac amyloid light-chain amyloidosis. Furthermore, the trials will study the potential of EGCG on preventing colon polyps in patients at high risk for recurrent colon adenoma and on reactivation of the Epstein–Barr virus in remission patients. More studies will investigate whether topical EGCG exerts an anticarcinogenic potential in patients with superficial basal cell carcinoma, whether gargling with EGCG inhibits influenza infections in teenagers and whether EGCG affects the resistance of insulin. Evidently, the trials will not study classic inflammatory disorder, though some of the mentioned diseases are linked with inflammatory processes (e.g., insulin resistance or Alzheimer’s disease). Nonetheless, it is probable that EGCG will experience an expansion of its indication in future.

### Quercetin

The flavonol, quercetin, chemically 2-(3,4-dihydroxyphenyl)- 3,5,7-trihydroxychromen-4-one, belongs to the family of polyphenols representing very extensively spread secondary plant metabolites. Quercetin is found in a variety of food like tea, capers, red onions, broccoli, berries, grapevines, and apples. Quercetin has been found to exert anti-mutagenic, anti-oxidative, anti-inflammatory, anticancer/chemopreventive, neuroprotective, antihypertensive, and blood glucose-lowering activities (Middleton et al., 2000). Quercetin activated various kinases which phosphorylated eukaryotic initiation factor 2, thus inhibiting cell translation (Ito et al., 1999). The mechanisms behind these actions are wide and have been characterized extensively. Quercetin scavenged nitrogen and reactive oxygen species (ROS), targets noticeable pro-inflammatory signaling pathways including MAPK, NF-κB and STAT1, and inhibits replication of many types of viruses and infectivity of target cells (Boots et al., 2008). The activity of COX-2 and iNOS was inhibited by quercitin by the suppression of AP-1, NF-κB and STAT-1 signaling in cytokine- or LPS-induced HUVECs and macrophages (Hamalainen et al., 2007). The expression of pro-inflammatory cytokines in calcium ionophore- and PMA-induced mast cells was attenuated by quercitin. Moreover, the TNF-α-stimulated NF-κB recruitment to pro-inflammatory gene promoters was also suppressed by quercitin in murine intestinal epithelial cells (Ruiz et al., 2007; Park et al., 2008). The TNF-α- or PMA-induced expression of ICAM-1 in human endothelial cells was decreased by quercitin (Kobuchi et al., 1999). The LPS-stimulated NF-κB and nitrite oxide production was also inhibited by quercitin in mice.

A number of studies on inflammatory parameters in humans have been performed in the past few years: one clinical trial estimated the effect of quercetin on biomarkers of inflammation depending on the apo-lipoprotein E genotype of healthy men. Though the risk factors of cardiovascular disease were improved, quercetin exerted a slight pro-inflammatory effect (Pfeuffer et al., 2013). After repeated sprint exercise, quercetin did not affect the levels of the pro-inflammatory cytokine IL-6 (Abbey and Rankin, 2011). Quercetin decreased the markers of inflammation (IL-8 and TNF-α) in sarcoidosis patients (Boots et al., 2011). Quercetin did not change phagocytosis activity, granulocyte oxidative burst or blood leukocyte subsets, TNF-α or IL-6 plasma levels in healthy females (Heinz et al., 2010). It is worth mentioning that no trial reports on the improvement of clinical parameters of inflammatory diseases (incidence and severity). Ten trials are registered on http://www.clinicaltrials.gov/ that will use pure quercetin. Regarding inflammatory disorders, quercetin will be analyzed in two-phase 1–2 trials for its dose-response relationship and safety in chronic obstructive pulmonary disease. For diabetes, quercetin will be tested in a phase 2 trial for its effect on blood vessel function and blood glucose in type 2 diabetes. Quercetin will also be administered in obese patients (with or without type 2 diabetes) to investigate its action on glucose absorption. In study related to cancer, it will be studied whether quercetin can prevent prostate cancer and whether it controls levels of prostate-specific antigen. Additionally, quercetin will be administered in children with Fanconi anemia (pharmacokinetics and safety). Therefore, quercetin will go through interesting studies that may lead to insightful development of knowledge regarding its clinical efficacy. Though, inflammatory diseases are not the main areas of current research.

### Colchicine

The tropolone derivative, colchicine, chemically (N-[(7S)-1,2, 3,10-tetramethoxy-9-oxo-6,7-dihydro-5H-benzo(a)heptalen-7] -ylacetamide), is the main alkaloid of *Colchicum autumnale* (family: Colchicaceae), generally called meadow saffron or autumn crocus. The extracts of this plant have been used against gout attacks for centuries. The US FDA has approved colchicine for the prevention and treatment of familial mediterranean fever and acute gout flares. To obtain this approval, the applying company provided new clinical data and was thus given an exclusive marketing agreement, 7 years for familial Mediterranean fever and 3 years for the indication gout (orphan drug status). The mechanisms of action of colchicine are extensively studied: the molecular target was recognized, the binding spot was accurately characterized, and the biological consequences of damaging microtubule dynamics were examined; complete reviews summarizing these results are available (Bhattacharyya et al., 2008; Nuki, 2008; Stanton et al., 2011). In 1960s, colchicine played a key role for the initial characterization of tubulin subunits and microtubules (Borisy and Taylor, 1967).

In spite of this vast knowledge, researchers have recently conducted numerous trials to expand its fields of application. Several trials in the field of inflammation-associated pathologies with positive outcome: colchicine was tested as an adjunct treatment against acute pericarditis. In an open-label, single-center randomized trial, colchicine addition to glucocorticoids or aspirin traditional treatment reduced the reappearance to half, after early occurrence of acute pericarditis. In a placebo controlled, double-blind, randomized, multicenter trial, colchicine, when added to traditional anti-inflammatory therapy, substantially decreased the rate of recurrent or incessant pericarditis (Imazio et al., 2005a,b, 2011, 2013), to prevent atrial fibrillation after radiofrequency ablation (Deftereos et al., 2012) and for the prevention of post-pericardiotomy syndrome (Imazio et al., 2010). These well-performed and large studies will definitely affect pharmacotherapy guidelines. And the field is still active: 19 clinical trials that are in the recruiting phase, are listed on http://www.clinicaltrials.gov/. They aim to study the action of colchicine primarily in the areas of nephrology and cardiology, for instance, in myocardial infarction, for the prevention of post-pericardiotomy syndrome, or in diabetic nephropathy-all related to inflammatory processes. Colchicine will be investigated to determine its effects on metabolism in adults who are overweight but have not yet developed diabetes. As the number of diseases with an inflammatory component is large, one might wonder that colchicine will further stay an interesting, not yet fully used drug.

### Capsaicin

Capsaicin, chemically (E)-*N*-[(4-hydroxy-3-methoxyphenyl) methyl]-8-methylnon-6-enamide, is a hydrophobic alkaloid found in chili peppers (*Capsicum* species; Solanaceae) and accounts for the characteristic spiciness/pungency of the fruits of the genus. It has been used in traditional medicine as a counter-irritant and topical rubefacient to relieve pain of joints and muscles. Recently, an 8% capsaicin cutaneous patch has been approved by the EU for use in non-diabetic adults against neuropathic pain and in US against neuropathic pain linked with post-herpetic neuralgia. The research on capsaicin resulted in the discovery of transient receptor potential channel vanilloid subfamily member 1 (TRPV1), which is the direct target of capsaicin (Caterina et al., 1997). TRPV1 is a positively charged indiscriminate channel mainly located in nociceptive neurons with high preference for Ca^2+^. It is activated by physical and chemical stimuli, like certain inflammatory mediators, capsaicin, low pH and heat (O’Neill et al., 2012). Extended activation of TRPV1 by capsaicin was found to cause desensitization and, therefore, decreased pain sensation (Haanpaa and Treede, 2012). Apart from pain, numerous studies in arthritic rats also discovered that capsaicin could inhibit paw inflammation (Joe et al., 1997; Park et al., 2000) and ethanol-induced inflammation of gastric mucosa in rats. Furthermore, capsaicin was found to inhibit NF-κB pathway, iNOS expression and COX-2 activity in the macrophages in a TRPV1-independent manner (Kim et al., 2003). Regarding inflammation, capsaicin was studied in a latest review by the Cochrane Collaboration: topical capsaicin was discovered to be ineffective against osteoarthritis (Cameron and Chrubasik, 2013). In comparison, one meta-analysis found adequate evidence to report that capsaicin was effective in the management of osteoarthritis, though the authors reported the lacking of randomized clinical trials (De Silva et al., 2011). As far as future therapeutic enhancements are concerned, 24 clinical trials are currently recruiting or are planned, as listed on http://www.clinicaltrials.gov/. Majority of these studies use capsaicin as a model substance to induce pain or as a diagnostic tool. Studies that examine the therapeutic potential of capsaicin study its potential as pre-emptive analgesic in patients going through amputation of a limb and its effect on neuropathic pain from critical ischemia (mainly in feet and hands) and on chronic pain from artificial arteriovenous fistulae in end-stage renal failure patients. Besides, capsaicin will be tested against the decreased swallow response in stroke patients with or opharyngeal dysphagia and against persistent pain after inguinal herniotomy. A study on the mechanism of action of capsaicin against idiopathic rhinitis will be carried out. No study will investigate the immunomodulatory effects of capsaicin. It can be concluded that, in comparison with neuropathic pain, the field of inflammation and capsaicin is not very cutting-edge and will stay on that level in the near future owing to lack of clinical trials.

### Andrographolide

Andrographolide is a diterpenoid lactone found in *Andrographis paniculata*. Chemically, it is (3-[2-[decahydro-6-hydroxy-5-(hydroxymethyl)-5, 8-dimethyl-2-methylene-1-napthalenyl] ethylidene] dihydro- 4-hydroxy-2(3*H*)-furanone). Diverse biological activities have been exhibited by andrographolide. Numerous immunomodulatory activities of andrographolide have been observed *in vitro* including reduction of IL-12, TNF-α, PGE_2_, NO, COX-2 and iNOS in microglia and macrophages (Maiti et al., 2006). The LPS-stimulated iNOS and COX-2 expression in RAW264.7 macrophages was inhibited by andrographolide. It also inhibited Akt and Erk ½ signaling, consequently inhibiting the chemotactic migration of macrophages on the site of inflammation (Tsai et al., 2004; Qin et al., 2006). In neutrophils, the production of ROS was inhibited by andrographolide (Shen et al., 2002). Andrographolide regulated the production of factors such as NK cells, IFN-γ, IL-2, and TNF-α. The administration of andrographolide resulted in improved expression of CD markers and production of TNF-α, therefore increasing the cytotoxic potential of lymphocytes (Rajagopal et al., 2003). Andrographolide inhibited IL-2, IFNγ and IL-6 expression, decreasing the cellular and humoral adaptive immune reaction in T cells. Andrographolide decreased the antigen-presenting potential of dendritic cells to T cells. In an ovalbumin-induced asthma rat model, the andrographolide administration decreased the serum immunoglobulin, IL-13, IL-4, IL-5, and Th2 cytokine. Andrographolide decreased migration and invasion, endothelial cell proliferation and adhesion molecule ICAM-1, signifying its role in angiogenesis (Chiou et al., 2000). The functions of NF-κB were inhibited by a number of immunomodulatory responses and andrographolide inhibited NF-κB binding to DNA and hence decreasing pro-inflammatory proteins expression like iNOS and COX-2 (Wang et al., 2004; Xia et al., 2004; Hidalgo et al., 2005; Iruretagoyena et al., 2005). Andrographolide down regulated COX-2 and iNOS gene expression by inhibiting NF-κB and signal transducer and activator of transcription-3 (STAT3) expression via suppressing the expression of suppressor of cytokine signaling 1 and 3 (Lee et al., 2011). Zhang et al. (2014) conducted a study to determine the effect of andrographolide on insulinoma tumor growth. Andrographolide was found to inhibit the progression of insulinoma tumor by targeting the TLR4/NF-κB signaling pathway (Zhang et al., 2014).

In spite of this vast knowledge, researchers have recently conducted numerous trials to expand its fields of application. But most of the trials have been conducted by using the crude extract. A double blind study was conducted on 101 patients with moderately active Crohn’s disease to assess the efficacy and safety of aqueous ethanol extract of *A. paniculata.* At week 8 of treatment, a significant decrease in the mean C-reactive protein level was observed in extract treated group as compared with the placebo group (Sandborn et al., 2010). A phase II study in patients with mildly to moderately active ulcerative colitis demonstrated that the efficacy of the aqueous ethanol extract of *A. paniculata* was similar to that of slow release mesalazine (Tang et al., 2011). In contrast to the patients with mildly to moderately active ulcerative colitis receiving placebo, those treated with *A. paniculata* extract were more probable to attain clinical response (Sandborn et al., 2013). In a double blind against placebo controlled clinical trial, the standardized patented *A. paniculata* extract’s (Paractin^®^) effect was evaluated in chronic rheumatoid arthritis patients. A significant reduction in total grade of tender joints, number of tender joints, total grade of swollen joint, number of swollen joints was observed. Serological parameters and rheumatoid factor (C4 and IgA) of disease were found to be reduced (Burgos et al., 2009). Currently, five different studies related to the various actions of andrographolide could be found on http://www.clinicaltrials.gov/. Two trials are registered on http://www.clinicaltrials.gov/ that will use pure andrographolide. A double blind, interventional, randomized, and placebo controlled phase II trial will determine the safety, acceptability, and effectiveness of andrographolide in active rheumatoid arthritis-affected patients and effects on the immunological functions that effect chronic inflammation will also be determined. A study will be conducted to determine the efficacy of andrographolide in retarding the progression of brain atrophy in patients with progressive forms of multiple sclerosis. Besides, the efficacy and safety of andrographolide will also be evaluated for the treatment of colorectal cancer.

### Genistein

Genistein, chemically 4,5,7-trihydroxyisoflavone, is a naturally occurring phytoestrogen, present in soy and soy-derived items. Genistein is a renowned tyrosine kinase inhibitor. *In vitro*, genistein inhibited IL-1b/IFNγ-induced COX-2 and iNOS expression and in addition to producing PGE_2_ and NO in human islets that may prevent pathogenesis of diabetes and improve insulin resistance (Corbett et al., 1996). Like other topoisomerase inhibitors, genistein induced apoptosis (Mccabe and Orrenius, 1993). Genistein strongly inhibited angiogenesis, exerted inhibiting effect on proliferating cells (Fotsis et al., 1995). Genistein regulates vascular function and protects from atherosclerosis (Si and Liu, 2007). The administration of genistein in human brain microvascular endothelial cells and HUVECs significantly inhibited the expression of TNF-α-induced cell adhesion molecule CD106 and CD62E, and monocyte adhesion (Lee and Lee, 2008). Genistein also reduced the interaction between endothelial cells and monocyte via activation of PPARs that reduced monocyte adhesion in culture cells and animals (Kim et al., 2004; Chacko et al., 2007). The LPS-stimulated secretion of MCP-1 from macrophages was inhibited by genistein that resulted in decreased migration of monocyte *in vitro* (Nagarajan et al., 2008). Genistein inhibited LPS-induced expression of nitro tyrosine protein and iNOS in vascular tissue that inhibits vascular alterations and hypotension *in vivo* (Bermejo et al., 2003). Genistein exerted potential effect on chronic colitis, neurodegenerative diseases, rheumatoid arthritis, metabolic disorders, and diabetes by modulating inflammatory response. For example, genistein inhibited production of pro-inflammatory molecules called as markers of cartilage catabolism (HC gp-39, IL-1b, and NO) in LPS-induced human chondrocytes (Hooshmand et al., 2007). Genistein modulated Th1-predominant immune response by increasing IL-4 production and suppressing the secretion of IFN-γ in a collagen-induced rheumatoid arthritis rat (Wang et al., 2008). NAFLD is an obesity-linked fatty liver illness sourced by pro-inflammatory cytokines and TNF-α and results in increase fatty acid uptake and the dysfunction of hepatocytes. Genistein reduced the high fat diet-induced steatohepatitis by improving liver function and decreasing plasma TNF-α level in rat (Yalniz et al., 2007). Moreover, genistein reduced LPS-stimulated dopamine uptake loss in rat mesencephalic neuron glial cells by decreasing production of TNF-α and NO and microglia activation (Wang et al., 2005) this might protect the pathogenesis of Parkinson’s disease caused by dopaminergic neuron injury. The increase of astrocytes at Ab deposition sites is the initial neuropathological fluctuation that starts inflammation in Alzheimer’s disease. The production of Ab-stimulated inflammatory mediators IL-1β, TNF-α, COX-2, and iNOS in astrocytes was decreased by genistein treatment, probably by PPARs’ activation (Valles et al., 2010). Likewise, oral administration of genistein decreased TNBS-stimulated chronic colitis by inhibition of COX-2 mRNA and protein expression together with the colonic myeloperoxidase (MPO) activity in rat that applies useful anti-inflammatory effects in the treatment of IBD (Seibel et al., 2009).

Genistein has undergone clinical trials for a variety of diseases like type 2 diabetes mellitus, menopause, osteopenia, and various types of cancer. The most noticeable diseases for which trails had been conducted were cancer. Genistein is undergoing clinical trials for chemopreventive and chemotherapeutic purposes in various cancers. Currently 11 open clinical trials have been in recruiting or planned according to http://www.clinicaltrials.gov. The main field of interest is cancer (colon cancer; rectal cancer; colorectal cancer, prostate cancer, bladder cancer, adenocarcinoma, pancreatic adenocarcinoma) followed by osteoporosis, hypercholesterolemia, Alzheimer’s disease and menopause.

## Conclusions and Future Prospects

Natural products and folklore medicines are the main contributors of the leads in the design and development of therapeutic agents. Several plant derived compounds have been identified over the years for their immunomodulatory characteristics. Numerous illnesses can be alternatively treated by immunomodulation using medicinal plants, instead of chemotherapy. The discovery and isolation of more specific immunomodulatory agents from plant origin possesses potential to counteract the side effects and high cost of synthetic compounds. This review highlights the significance of medicinal plants as producers of immunomodulatory molecules of very varied chemistries with possible uses in animal and human health. The challenges encountered by the application of plant-derived immunomodulators need to be addressed. Though, the path from traditional medicines to western pharmaceutical practices is not always easy. The inconsistency of responses of phytomedical practices can be clarified in terms of the typically strong reliance of plant secondary metabolite profiles on environmental signals that can disturb reproducibility of results with extracts. This can be decreased if the principles of standardization of extracts and enriched fractions are thoroughly applied.

In most of studies conducted to determine the effect on immune system, no adequate microbial contamination control protocols were applied. Researchers report that the microbial endotoxin can change the parameters of immune system. Therefore, appropriate precautions must be taken to counter the microbial contamination. An additional task is the classification of plant-derived immunomodulatory agents in a specific class or among classes as per inherent risk. This classification of novel plant derived immunomodulatory agents by level of risk can be endeavored from collective knowledge of meta-analyses of clinical trials, national registries and physicians. Another significant constraint with natural products is inadequate quantities, needed for development and clinical use. So, the development of novel isolation techniques to improve the amount for pharmaceutical applications needs more attention of researchers. A major clinical restraint of these substances is the low bioavailability. Nanotechnology and other delivery strategies are being applied to enhance their efficacy when administered to humans. Lastly, there is a deficiency of standard checking and quality control procedures to assure the efficacy and quality of the plant derived medicinal products for prospective pharmaceutical applications. A few plant derived compounds, including polysaccharides, are extremely diversified in terms of molecular weight and structure; therefore, it is challenging to produce the similar quality in every batch. Accumulative requirement for these plant-derived products would motivate improvement to overwhelming these obstacles for reaching market.

## Conflict of Interest Statement

The authors declare that the research was conducted in the absence of any commercial or financial relationships that could be construed as a potential conflict of interest.
