# Corrigendum: Plant-derived immunomodulators: an insight on their preclinical evaluation and clinical trials

**DOI:** 10.3389/fpls.2018.01178

**Published:** 2018-08-13

**Authors:** Ibrahim Jantan, Waqas Ahmad, Syed Nasir Abbas Bukhari

**Affiliations:** Drug and Herbal Research Centre, Faculty of Pharmacy, Universiti Kebangsaan Malaysia, Kuala Lumpur, Malaysia

**Keywords:** immunomodulation, curcumin, resveratrol, epigallocatechol-3-gallate, quercetin, colchicine, capsaicin

In the original article **“**Fürst and Zündoft, 2014**”** was not cited in the article. The citation has now been inserted in section Immunomodulators, Paragraph 3 and should read:

“Recently the clinical potential of six plant-derived anti-inflammatory compounds: curcumin, colchicine, resveratrol, capsaicin, epigallocatechin-3-gallate (EGCG), and quercetin has been highlighted Fürst and Zündoft (2014). The present review will give an overview of these widely investigated plant-derived compounds including andrographolide and genistein, which have exhibited potent effects on cellular and humoral immune functions in pre-clinical investigations and will highlight their clinical potential.”

The citation has now been inserted in section Curcumin, Paragraph 2 and should read:

“However, Fürst and Zündoft (2014) suggested that these are preliminary clinical trials which are frequently too weak and of low quality to draw a conclusion due to the low number of enrolled patients, which normally ranges from 10 to 30. As suggested by the authors, more operationally thorough and serious randomized controlled trials are required to evaluate the compound as an effective and safe agent for human use. It is worth mentioning that curcumin suffers from its low bioavailability, though substantial improvement has been made to address this issue via chemical and technological methods (Anand et al., 2007).”

The authors apologize for this error and state that this does not change the scientific conclusions of the article in any way.

The original article has been updated.

## Conflict of interest statement

The authors declare that the research was conducted in the absence of any commercial or financial relationships that could be construed as a potential conflict of interest.
